# Advancing cell-free DNA as a biomarker of damage to heart caused by ionizing radiation

**DOI:** 10.1093/jrr/rraf022

**Published:** 2025-04-30

**Authors:** Erin Wallisch, Aoy Tomita-Mitchell, Huan-Ling Liang, Aniko Szabo, Marek Lenarczyk, Anne Kwitek, Jennifer R Smith, Monika Tutaj, John E Baker

**Affiliations:** Division of Congenital Heart Surgery, Medical College of Wisconsin, 8701 Watertown Plank Road, Milwaukee, WI 53226, USA; Division of Congenital Heart Surgery, Medical College of Wisconsin, 8701 Watertown Plank Road, Milwaukee, WI 53226, USA; Division of Congenital Heart Surgery, Medical College of Wisconsin, 8701 Watertown Plank Road, Milwaukee, WI 53226, USA; Data Science Institute, Medical College of Wisconsin, 8701 Watertown Plank Road, Milwaukee, WI 53226, USA; Division of Congenital Heart Surgery, Medical College of Wisconsin, 8701 Watertown Plank Road, Milwaukee, WI 53226, USA; Radiation Biosciences Laboratory, Medical College of Wisconsin, 8701 Watertown Plank Road, Milwaukee, WI 53226, USA; Department of Physiology, Medical College of Wisconsin, 8701 Watertown Plank Road, Milwaukee, WI 53226, USA; Department of Physiology, Medical College of Wisconsin, 8701 Watertown Plank Road, Milwaukee, WI 53226, USA; Department of Physiology, Medical College of Wisconsin, 8701 Watertown Plank Road, Milwaukee, WI 53226, USA; Division of Congenital Heart Surgery, Medical College of Wisconsin, 8701 Watertown Plank Road, Milwaukee, WI 53226, USA; Radiation Biosciences Laboratory, Medical College of Wisconsin, 8701 Watertown Plank Road, Milwaukee, WI 53226, USA

**Keywords:** cell-free DNAbiomarker, nucleus, mitochondria, *Gapdh*, 12S rRNA

## Abstract

Exposure to diagnostic and therapeutic radiation introduces risks for development of diseases later in life by causing DNA damage in cells. Currently, there is no clinical method for determining exposure risk caused by radiation toxicity to DNA. Cell-free DNA (cfDNA), a marker of DNA damage, is currently used to assess risk for long-term effects following organ transplantation, surgery and inflammation. The goal of our proposed study is to develop cfDNA as an early biomarker for assessing risk for cardiovascular disease and cancer from radiation exposure so that strategies to mitigate the damaging effects of medical radiation can be assessed. Hearts from male and female WAG/RijCmcr rats (*n* = 6–10/group) were exposed to increasing doses of X-radiation (50 mGy and 3.5 Gy). Blood was collected prior to and after (15 minutes–96 hours) irradiation, and cell-free plasma was prepared. Primers and probes were designed for quantitative analysis of sequences of mitochondria (12S rRNA) and nuclear (*Gapdh*) cfDNA present in rat plasma using quantitative reverse transcription polymerase chain reaction (RT-qPCR). Exposure of hearts to radiation increased nuclear and mitochondrial cfDNA in a dose-dependent manner. Three point five grays from X-radiation increase cfDNA for *Gapdh* in plasma after 1 hour with a 15.8-fold increase (*P* < 0.001) after 6 hours. The earliest time nuclear and mitochondrial cfDNA increases were detected in plasma was at 60 minutes following exposure to 3.5 Gy. cfDNA has potential to advance as a biomarker of exposure to medical doses of radiation in patients.

## INTRODUCTION

Ionizing radiation contains sufficient energy to displace electrons and break chemical bonds. When traversing tissue, ionizing radiation interacts either directly with DNA molecules by displacing electrons, which leads to ionization, or indirectly by transferring part of its energy to one of the electrons in the traversed molecule, which, in turn, ionizes surrounding molecules such as DNA. Given the high content of water in cells, this ionization induces the production of reactive oxygen species that can result in DNA damage. Unrepaired DNA damage may lead to mutation and initiation of carcinogenesis and cardiovascular disease.

Ionizing radiation is widely used for cancer therapy and diagnostic imaging, which directly exposes the heart to radiation [1–3]. Exposure to high-dose ionizing radiation during radiotherapy can damage the heart. Radiotherapy for breast cancer that delivered a mean dose of 4.9 Gy to the heart increased the risk of a subsequent coronary event at a rate of 7.4% per gray in a linear fashion [4]. Epidemiological studies support a causal association between high-dose radiation exposure for the two main types of circulatory disease: ischemic heart disease and cerebrovascular disease [5]. Cardiovascular disease risk in the low dose range (<100 mGy), representative of doses that patients receive from medical diagnostic exposures to radiation, is not well understood [6].

Determining the health implications of ionizing radiation at the organ level would require an assay specific to radiation exposure. Currently, there is no clinical method for determining treatment risk caused by radiation toxicity to DNA and for personalizing the radiation dose during treatment. The most closely related technologies are non-personalized biodosimetry for total body irradiation, such as lymphocyte counts, chromosomal aberrations, radical traps, cytokine profiles and gammaH2AX measurements [7, 8]. Urinary 8-hydroxy-2′ deoxyguanosine levels, a biomarker of radiation-induced DNA damage in children undergoing cardiac catheterization, increase 24–48 hours after exposure to 70 mGy [9]. Cytogenic analysis of dicentric chromosomes is the gold standard for biological dosimetry [10]; however, sensitivity of the assay is limited to doses >100 mGy. Peripheral blood reticulocytes can be used for radiation dosimetry [11]. For monitoring DNA damage at doses >1 mGy, gammaH2AX immunofluorescence microscopy has evolved as a more sensitive and appropriate method [10]. These methods require the use of specialized techniques that can be expensive and time consuming. Taken together, these findings suggest the need for development of better biomarkers of radiation exposure for children with congenital heart disease and adults with acquired heart disease to understand risks from exposure to radiation and to predict health outcomes.

Genomic DNA (gDNA) in healthy cells is normally confined within the nucleus of cells. However, gDNA is released into the circulation as cell-free DNA (cfDNA) during cell aging, where cells undergo programmed death or apoptosis or during early disease states [12]. These circulating cfDNAs are present at very low concentrations in healthy individuals because of their homeostatic ability to efficiently eliminate them from the circulation [13]. However, cfDNA levels increase in infection and markedly increase in conditions characterized by significant inflammation or traumatic tissue injury [14].

Radiation-induced damage to DNA can either be repaired and cells function normally, be imperfectly repaired and cells function abnormally, or cells may die because of this damage, either directly or, as is more usual, indirectly at cell division. Much attention has been focused on DNA since DNA damage plays an important role in the evolution of multiple disorders and aging processes. Thus, DNA damage has potential to be used as a biomarker to quantify radiation exposure and assess its possible long-term effects, such as increased risk for cancer and cardiovascular disease.

In patients who have been exposed to medical radiation during diagnostic and therapeutic procedures, radiation exposure may result in high levels of gDNA being released into circulation. However, there is currently no available method for an efficient, reliable and inexpensive means for detecting radiation exposure in these individuals. Accordingly, there is an urgent need for methods and/or biomarkers useful for detecting radiation exposure to cells, especially based on gDNA released into circulation upon pathophysiological insults. Total and mitochondrial cfDNA levels are elevated in cardiologists during occupational exposure to medical radiation [15]. CfDNA is used as a marker of DNA damage to assess risk for long-term effects following organ transplantation [13, 16, 17] and cardiac surgery [18].

We reasoned that measuring nuclear and mitochondrial cfDNA present in the circulation would allow for the development of a biomarker of damage to the heart from radiation. In this study, we targeted a single-copy nuclear gene to measure nuclear cfDNA (*Gapdh*). In addition, a mitochondrial target was used to measure both long and short mitochondrial cfDNA fragments of 12S rRNA. Mitochondria are present in multiple copies per cell (on average 60 per cell) and are particularly enriched in cardiomyocytes (up to 400 copies per cell). Therefore, the advantages of a mitochondrial cfDNA assay are that mitochondrial cfDNA may be particularly sensitive to cardiomyocyte death and would require a reduced draw volume, a desirable characteristic for pediatric or neonatal applications. Furthermore, because cfDNA is typically found at fragment lengths of ~168 bps in length, we were interested in assessing mitochondrial cfDNA lengths. Therefore, primers specific for different lengths with a shared TaqMan probe were designed that targeted a region of the rat mitochondrial DNA sequence that was highly conserved across over 100 unique rat strains or substrains [19, 20]. Our objective was to develop measurements for cfDNA in blood as a biomarker of radiation-induced damage at doses relevant to pediatric cardiac catheterization and to adult radiotherapy for cancers in the thorax such as breast, lung and esophagus that would be predictive of clinical outcomes and radiogenic disease. Our long-term goal is the development of early and non-invasive detection measures of radiation-induced damage, quantitative measurement of that radiation-induced damage and the development of a kit that is quick and easy to use.

The hypothesis tested was that cfDNA can be utilized as a biomarker of radiation-induced damage. The objective of our study was to determine the levels of nuclear and mitochondrial cfDNA in rat plasma before and after local exposure of the heart to 50 mGy and 3.5 Gy of X-radiation.

## MATERIALS AND METHODS

### Experimental animals

This study was conducted in WAG/RijCmcr rats (RRID: RGD_2303640), an inbred strain with no pre-existing cardiac disease, sensitive to low- and high-linear energy transfer radiation-induced pathology [21, 22]. Rats were maintained and studied according to the protocol approved by the Institutional Animal Care and Use Committee of the Medical College of Wisconsin (protocol AUA996). Male rats at 5–6 weeks of age were selected to be representative of a pediatric population. To study the effects of radiation in an animal model closer to that of pediatric patients than in previous studies on the heart, rats were studied at the earliest age possible when they could live independently from their mother. This feature of the experimental design avoided any potential confounding effects arising from nutrition and care that could originate from the mother. WAG/RijCmcr rats are weaned at around 4 weeks of age so that by 5 weeks of age the offspring can exist independently. At 5 weeks of age, rats weigh 71 ± 3 g, 41% of the young adult body weight, with well-developed hearts, livers and lung, and thus, they are likely comparable to pre-pubescent humans [23]. Female rats at 11–12 weeks of age were selected as being representative of adults as they are sexually mature by this age.

### Sex as a biological variable

Male and female rats were studied as both sexes are exposed to medical radiation.

### Local heart irradiation

**Fig. 1:**
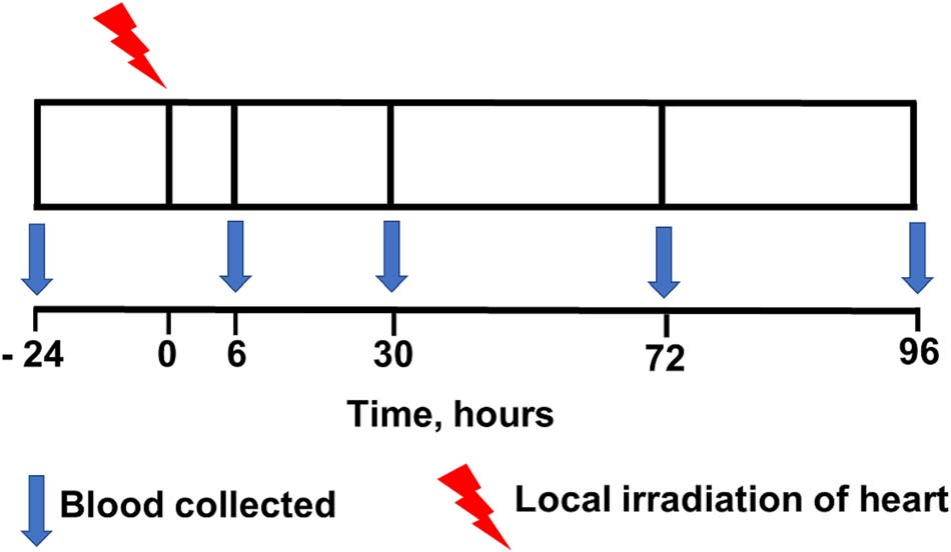
General experimental protocol. The heart was exposed to a single fraction of 50 mGy or 3.5 Gy of X-radiation. Peripheral blood was collected before (−24 hours) and after (+6, 30, 72 and 96 hours) irradiation and plasma prepared. In some studies, peripheral blood was collected at 15, 30 and 60 minutes after irradiation.

**Fig. 2:**
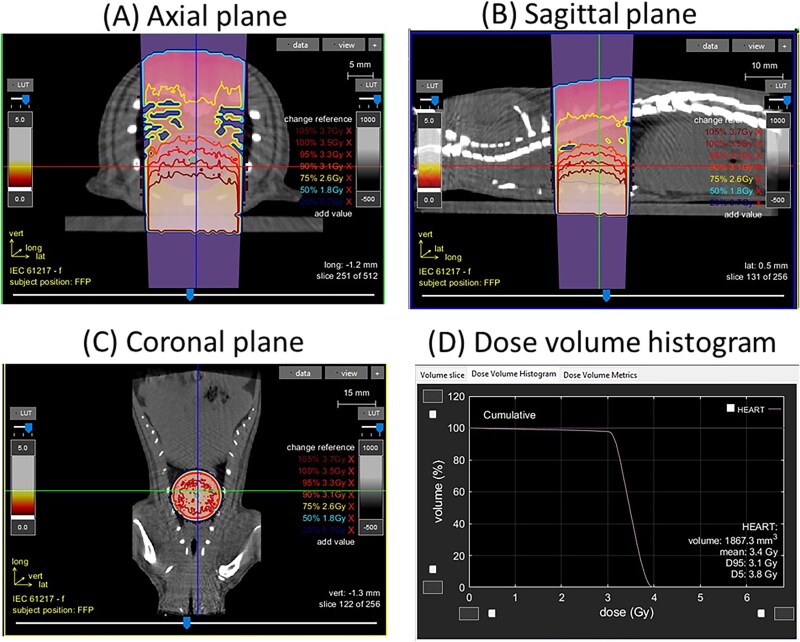
Image-guided localized heart radiation technique. Computed tomography images of a representative adult female rat at 11–12 weeks of age with a 1.5-cm-diameter collimator plan of 3.5 Gy encompassing the heart in equally weighted beams (two laterals) are shown in the axial (A), sagittal (B) and coronal (C) planes. The heart is located within the central circle of the image. (D) Dose volume histogram demonstrating dose to the heart.

The experimental protocol is shown in Fig. 1. Rats underwent local heart irradiation with a single fraction dose of X-radiation (50 mGy or 3.5 Gy) in the anterior posterior mode in the irradiation core at the Medical College of Wisconsin [24]. Anesthetized rats (3% isoflurane/air) were immobilized in a restraint jig to prevent movement during the period of irradiation. The anatomic location of the heart was confirmed using a PaxScan 2520 V Amorphous Silicon Digital X-ray Imager (Varian) attached to the X-RAD SmART irradiator unit to capture images of the exposed field [25] (Fig. 2). Hearts were irradiated using a SmART 320 kV orthovoltage X-ray system (Precision X-Ray, North Branford, CT). To deliver 50 mGy, the X-radiation system was operated at 225 kVp, 1 mA, with a 0.32 mm Cu filter using a 2 cm circular collimator with a dose-rate of 222 and 225 mGy/minute, respectively. To deliver 3.5 Gy, the X-radiation system was operated at 225 kVp, 20 mA, with a 0.32 mm Cu filter using a 2 cm circular collimator with a dose-rate of 4.56 Gy/minute. All irradiations were performed between 08:00 and 10:00 am.

### Blood collection and processing

Blood was collected from the rats *via* jugular venipuncture and collected at various time points in K_2_ ethylenediaminetetraacetic acid (EDTA) blood collection tubes (Becton Dickinson vacutainer spray-coated K_2_ EDTA tubes). Blood was collected prior to and following radiation, and plasma was prepared. EDTA is preferred over citrate and heparin as an anticoagulant, in part, because EDTA salts, in addition to protecting cfDNA by preventing release of cellular DNA through coagulative stress, inhibit *ex vivo* DNase activity [26]. Many studies have demonstrated stability of cfDNA within unspun whole blood samples without significant contamination by leukocyte gDNA when collected in K2-EDTA tubes and held at room temperature for 4–6 hours prior to plasma separation by centrifugation [13]. To effectively eliminate DNA from leukocytes in plasma, two sequential centrifugation steps were included [13]. Blood was centrifuged at 3000 rpm for 10 minutes at 4°C, the supernatant was removed and then centrifuged at 14 000 rpm for 10 minutes at room temperature. Plasma was then frozen at minus 80°C prior to cfDNA analysis. Rats were euthanized immediately after the last blood collection by intraperitoneal injection of pentobarbital (150 mg/kg) followed by pneumothorax *via* thoracotomy. DNA was extracted from samples of plasma using the QIAamp Circulating Nucleic Acid kit from Qiagen and Promega ReliaPrep Circulating Nucleic Acid Kit on the Tecan Freedom Evo. Both kits and instruments have been validated for equivalency in house [13].

### Nuclear and mitochondrial cfDNA assays

**Fig. 3:**
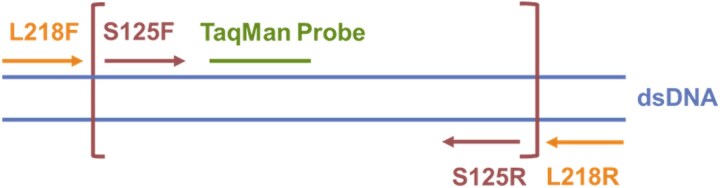
12S rRNA L218 and S125 PCR primer design for mitochondrial cfDNA probes. Forward and reverse primers of L218 and are indicated by orange text. Forward and reverse primers for S125 are indicated by brown text. L218 primers amplify long (218 bp) DNA fragments, while S125 primers amplify short (125 bp) DNA fragments.

TaqMan assays were developed to quantify nuclear and mitochondrial cfDNA targets in the plasma following radiation. Quantitative analysis of nuclear and mitochondrial cfDNA was performed by quantitative real-time PCR (qPCR). One primer set specific for the single-copy nuclear gene *Gapdh* was used to quantify nuclear cfDNA. This set was taken from Abdullaev *et al*. [27] and amplified an 80-bp fragment. The sequences were as follows: forward—5′-TGG CCT CCA AGG AGT AAG AAA C-3′; reverse—5′-GGC CTC TCT CTT GCT CTC AGT ATC-3′; and probe—FAM-CTG GAC CAC CCA GCC CAG CAA-MGBNFQ. To identify highly conserved rat mitochondrial genome targets [28], the mitochondrial genomes from >100 inbred rat strains or substrains available at the Rat Genome Database [19, 20] were aligned to the reference rat genome sequence (mRatBN7.2) and examined to identify invariant regions. Vcf files of mtDNA for the strains were obtained from Schlick *et al*. [28], and two primer sets were designed targeting a suitable region within the 12S ribosomal RNA in the mitochondrial rat genome (AJ005780) and confirmed with the RGD BED file. The first set was specific for a ‘long’ 218 bp (L218) fragment. The sequences were as follows: forward—5′-GCC ATC TTC AGC AAA CCC TA-3′; reverse—5′-GTG TGC GTA CTT CAT TGC TC-3′; and probe—FAM-TCC CAC TTC ATT GGC TAC ACC TTG AC-MGBNFQ. The second set was specific for a ‘short’ 125 bp (S125) fragment. The sequences were as follows: forward—5′- TCA CCA CCT CTT GCT AAT TCA G-3′; reverse—5′-TCT TCC CAC TTC ATT GGC TAC-3′; and probe—FAM- ATA CCG CCA TCT TCA GCA AAC CCT-MGBNFQ (Fig. 3). L218 primers were designed to bracket the S125 target. This means that the long primers only amplified the long sequence, while the short primers amplified long and short sequences. Primers and probes were designed using IDT software and synthesized by ThermoFisher Scientific. qPCRs were performed using the QuantStudio 7 Real-Time PCR System in a 10-μl volume containing 2 μl of Roche TaqMan Master Mix, 1 μl DNA solution, 0.2 μl ROX reference dye, 6.3 μl of nuclease-free water, and 900 nM of each primer and 250 nM of probe. PCR cycles for the nuclear assay were as follows: 2 minutes at 50°C, 10 seconds at 95°C, followed by 40 cycles of 95°C for 15 seconds and annealing and elongation at 60°C for 1 minute. PCR cycles for the mitochondrial 218 bp assay were as follows: 2 minutes at 50°C, 10 seconds at 95°C, followed by 40 cycles of 95°C for 15 seconds and annealing and elongation at 55°C for 1 minute. PCR cycles for the mitochondrial 125 bp assay were as follows: 2 minutes at 50°C, 10 seconds at 95°C, followed by 45 cycles of 95°C for 15 seconds and annealing and elongation at 57°C for 1 minute. Fluorescence values were analyzed and calculated by the QuantStudio 7 software using a standard curve dilution series of genomic DNA isolated from the buffy coat of rat whole blood and quantified by UV spectrophotometry. CfDNA content in each sample was evaluated in triplicate. Nuclear *(Gapdh*) cfDNA was expressed in nanograms per milliliter (ng/ml) of plasma. Mitochondrial cfDNA was determined using the same genomic DNA from rat buffy coat for a standard curve. To estimate mitochondrial copies, a calculation was performed assuming 6.3 pg/cell and 10 mitochondria per buffy coat cell.

### Biostatistical analysis

The amount of nuclear and mitochondrial cfDNA in plasma was summarized by mean and standard deviation at each timepoint. Nuclear cfDNA was measured in nanograms per milliliter (ng/ml), and mitochondrial cfDNA was measured in relative mitochondrial copies per microliter. Based on data by Abdullaev *et al*. [27], we expected the measurement variability to increase with the mean; thus, all analyses were performed on a logarithmic scale. This corresponds to studying multiplicative effects (i.e. fold changes) on the original scale. Fold change was calculated by dividing the value in the post-irradiation group by the value in the pre-irradiation group. For each measurement type, one-way repeated measures analysis of variance using a random intercept linear mixed effects model was used to model the change in cfDNA values over time, with focus on comparison to the pre-irradiation baseline. No adjustment for multiple testing was performed.

## RESULTS

There were no deaths over the 96-hour follow-up period. The total time the heart was exposed to 50 mGy X-radiation was 13.5 seconds and, for 3.5 Gy, it was 46.1 seconds.

### Nuclear and mitochondrial cfDNA levels in plasma at 6–96 hours after irradiation

A linear mixed-effect model with random sample effect was used on log-transformed measurements to evaluate the effect of time on nuclear and mitochondrial cfDNA levels in plasma. Estimates were back-transformed and interpreted as fold-change compared to time 0 (pre-radiation) values. Changes in cfDNA levels were dose dependent.

### Exposure of hearts to radiation increases nuclear cfDNA in plasma

**Fig. 4:**
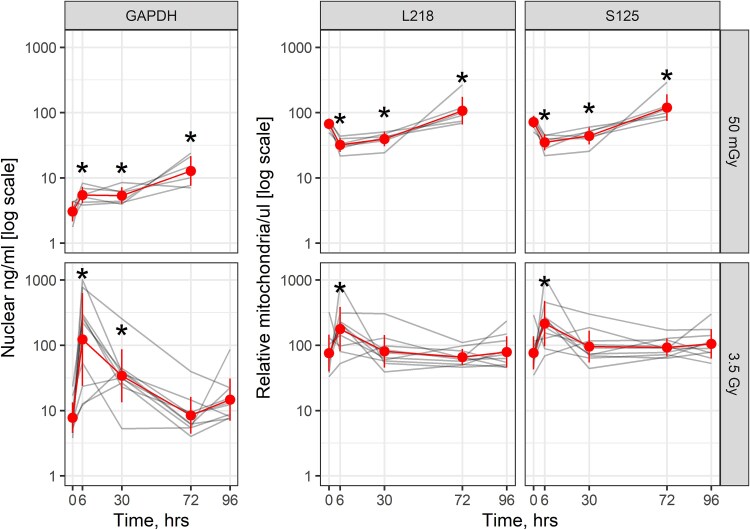
Changes in nuclear and mitochondrial cfDNA in plasma 6–96 hours after local heart irradiation. Red line represents the group average and standard deviation of the mean. Gray line represents values from an individual rat. Hearts from males (*n* = 6) were exposed to 50 mGy of X-radiation. Hearts from females (*n* = 10) were exposed to 3.5 Gy of X-radiation. Asterisks (*) represent a level of statistical significance compared with time 0 hours. Actual *P*-values are stated in the text.

In male WAG/RijCmcr rats, hearts (*n* = 6) were exposed to 50 mGy X-radiation, representative of a dose used for diagnostic radiation in patients with congenital heart disease [9]. Levels of nuclear cfDNA for *Gapdh* progressively increased over time in the plasma after exposure of the heart to 50 mGy X-radiation at 6 (*P* = 0.015), 30 (*P* = 0.019) and 72 hours (*P* < 0.001) by 1.78, 1.75 and 4.17-fold, respectively, compared with 0 hours. Exposure of female hearts (*n* = 10) to 3.5 Gy, representative of a dose used in therapeutic radiation [29–32], resulted in a significant (*P* < 0.001) 15.8-fold increase in nuclear cfDNA after 6 hours and a 4.40-fold increase at 30 hours (*P* < 0.001). Levels of nuclear cfDNA then declined after 72–96 hours (Fig. 4).

### Exposure of hearts to radiation changes mitochondrial cfDNA levels in plasma

**Fig. 5:**
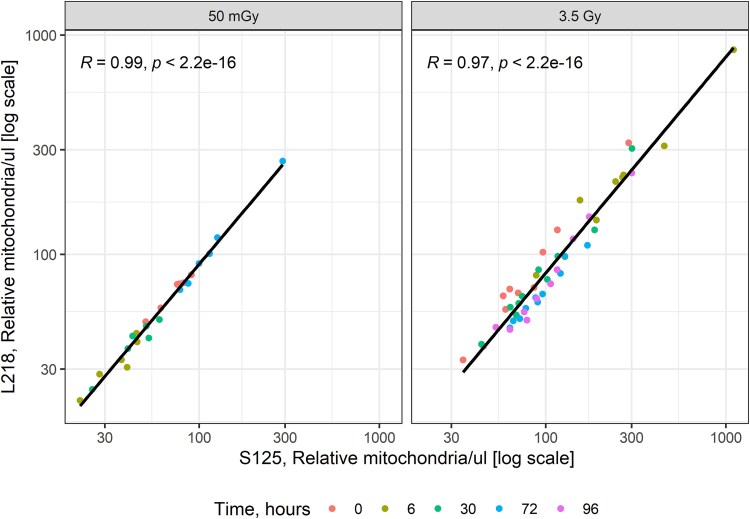
Scatter plot showing the correlation between L218 and S125 measurements 0–96 hours after local heart irradiation with 50 mGy and 3.5 Gy. Each point represents an individual data point. The solid line represents the linear regression line. The correlation coefficient *R* and *P*-value are indicated at the top of each figure for each dose. A significant positive correlation was observed between L218 and S125 measurements.

**Fig. 6:**
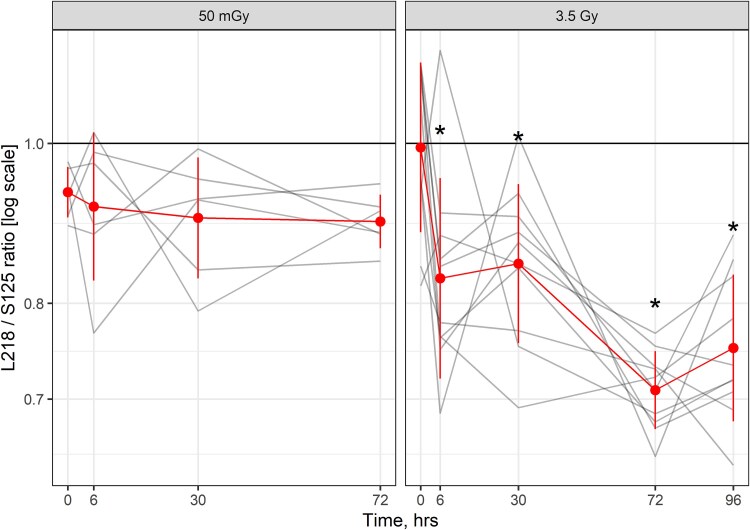
Ratio of long to short mitochondrial cfDNA in plasma 6–96 hours. Red line represents the group average and standard deviation of the mean. Gray line represents values from an individual rat. Asterisks (*) represent a level of statistical significance compared with time 0 hours. Actual *P*-values are stated in the text.

In male WAG/RijCmcr rats, the heart was exposed to 50 mGy X-radiation. Plasma levels of the L218 and S125 fragments of mitochondrial cfDNA for 12S rRNA were measured 6–96 hours after irradiation. L218 levels decreased in plasma 0.48-fold at 6 hours (*P* < 0.001) and 0.59-fold at 30 hours (*P* = 0.010). The levels of S125 decreased in plasma 0.49-fold at 6 hours (*P* = 0.001) and 0.61-fold at 30 hours (*P* = 0.018). L218 and S125 levels then increased at 72 hours by 1.59- (*P* = 0.020) and 1.66-fold (*P* = 0.016), respectively, compared with 0 hours (Fig. 4). Exposure of female hearts to 3.5 Gy increased levels of the long and short fragments of mitochondrial 12S rRNA at 6 hours by 2.34- (*P* = 0.003) and 2.81-fold (*P* < 0.001), respectively. Levels of the long and short fragments declined 30–96 hours after irradiation. Values for the L218 and S125 in plasma over 0–96 hours were then correlated. There was a strong positive correlation between the two assays for 50 mGy and 3.5 Gy (Fig. 5). We then evaluated the mitochondrial assays for sensitivity by comparing the ratios of the L218 and S125 mitochondrial fragments for the two doses of radiation. In male hearts exposed to 50 mGy, there was no decrease in the L218/S125 ratio over the period 6–72 hours. There was no sample available at 96 hours. In female hearts exposed to 3.5 Gy, the L218/S125 ratio decreased at 6, 30, 72 and 96 hours (*P* < 0.001) (Fig. 6).

### Nuclear and mitochondrial cfDNA levels in plasma 15–60 minutes after irradiation with 3.5 Gy

**Fig. 7:**
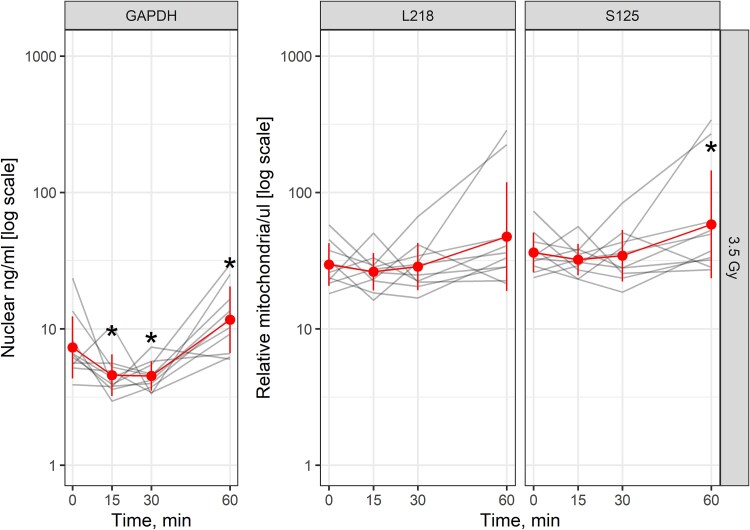
Changes in nuclear and mitochondrial cfDNA in plasma 15–60 minutes after local heart irradiation. Red line represents the group average and standard deviation of the mean. Gray line represents values from an individual rat. Hearts from females (*n* = 10) were exposed to 3.5 Gy of X-radiation. Asterisks (*) represent a level of statistical significance compared with time 0 hours. Actual *P*-values are stated in the text.

To determine the earliest time after radiation that changes in cfDNA levels in plasma are present, female hearts (*n* = 10) were exposed to 3.5 Gy and blood collected after 15, 30 and 60 minutes. The levels of *Gapdh* decreased 0.63-fold at 15 minutes (*P* = 0.023) and 0.62-fold at 30 minutes (*P* = 0.020) compared with 0 minutes. *Gapdh* levels then increased at 60 minutes by 1.60-fold (*P* = 0.023). L218 levels were unchanged 15–60 minutes after irradiation. S125 levels were unchanged at 15–30 minutes after irradiation and increased at 60 minutes (*P* = 0.041) by 1.61-fold (Fig. 7).

**Fig. 8:**
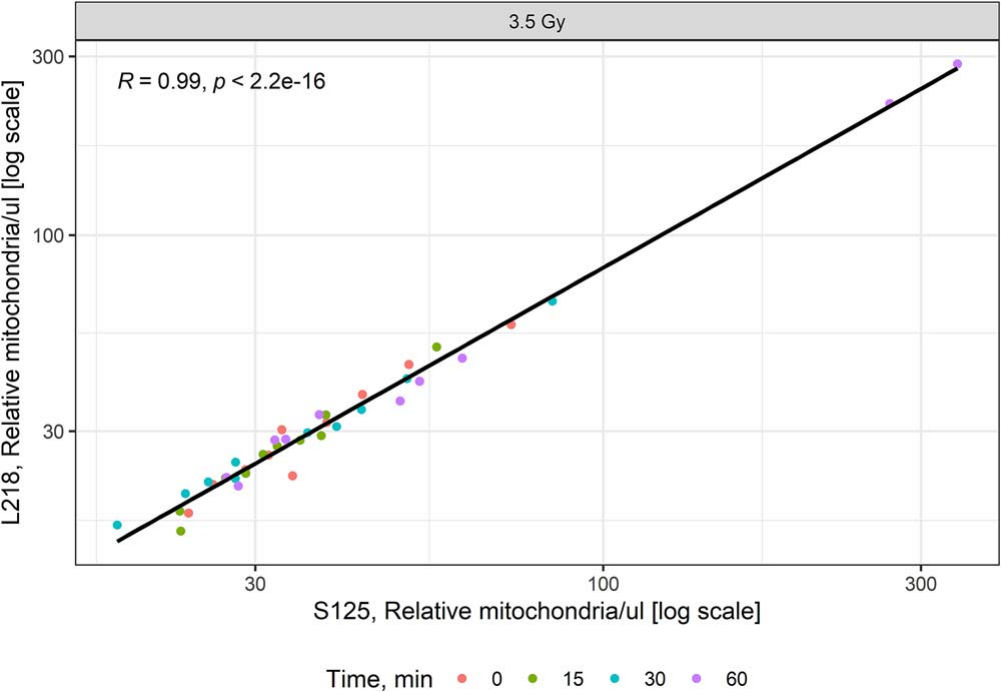
Scatter plot showing the correlation between L218 and S125 measurements 0–60 minutes after local heart irradiation with 3.5 Gy. Each point represents an individual data point. The solid line represents the linear regression line. The correlation coefficient *R* and *P*-value are indicated at the top of each figure for each dose. A significant positive correlation was observed between L218 and S125 measurements.

**Fig. 9:**
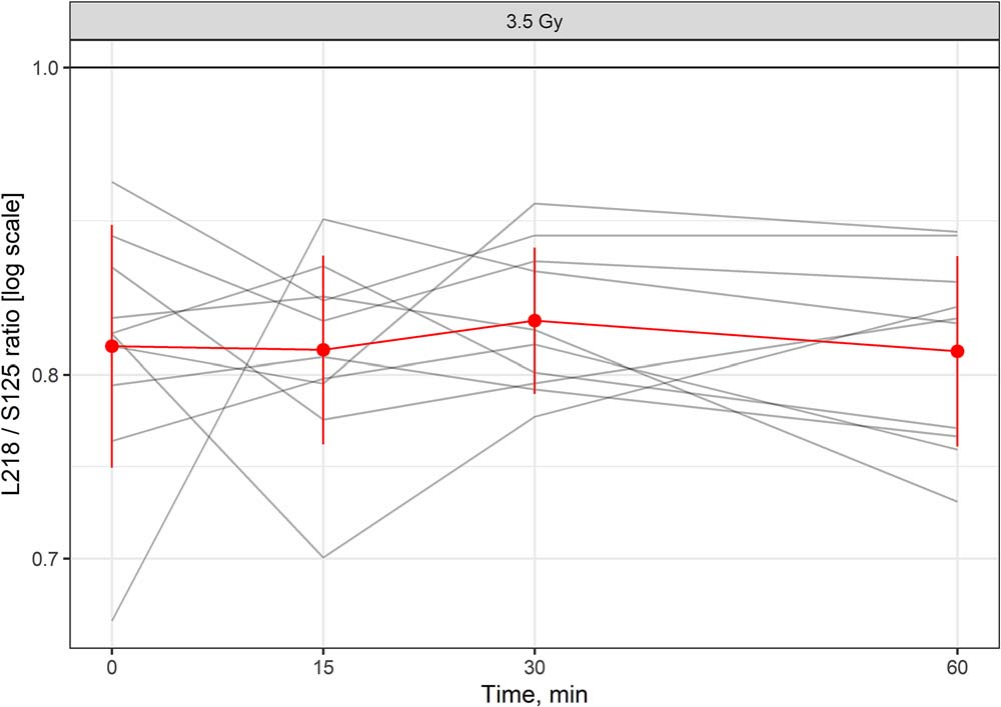
Ratio of long to short mitochondrial cfDNA 0–60 minutes after local heart irradiation. Red line represents the group average and standard deviation of the mean. Gray line represents values from an individual rat.

Values for L218 and S125 in plasma over 0–60 minutes were then correlated. There was a strong positive correlation between the two assays for 3.5 Gy (Fig. 8). We evaluated the mitochondrial assays for early sensitivity by comparing the ratios of the L218 and S125 mitochondrial fragments following exposure to 3.5 Gy. In hearts exposed to 3.5 Gy, there was no decrease in the L218/S125 ratio over the period 15–60 minutes (Fig. 9).

## DISCUSSION

Currently, there is no clinical method for determining risk of radiation toxicity to the heart in a patient receiving radiotherapy for a tumor within the thorax. To directly address this need, the current study establishes a new assay specific to early radiation-induced damage to the heart in a preclinical model designed to inform treatment risk for an individual patient. The findings of the present study establish our technical capability to: (i) quantify the levels of nuclear and mitochondrial cfDNA in rat plasma from multiple inbred strains, (ii) show these cfDNAs are present in non-irradiated rats in both biological sexes and (iii) describe a dose-dependent increase in plasma levels for nuclear and mitochondrial cfDNA in response to exposure of the heart to doses of radiation relevant to diagnostic and therapeutic radiation at 6–96 hours after exposure.

Biomarker measurements are typically made within hours after exposure to radiation to capture early biological changes. The findings of the present study indicate an initial decrease in biomarker levels for both doses of radiation followed by an increase. Possible reasons for this initial decrease in cfDNA may be radiation-induced inhibition of cfDNA release pathways in healthy heart cells, degradation by DNases present in the blood, renal excretion and uptake by macrophages resident in the kidney and liver [33–35]. An initial decrease in a candidate biomarker after exposure to radiation is not specific to nuclear or mitochondrial cfDNA. miR-150 exhibits a dose- and time-dependent (24- and 48-hour post-radiation) decrease after exposure to 1–8 Gy radiation [36]. This suggests a biphasic time course of biomarker release that needs further study. The higher radiation dose resulted in faster and more pronounced biomarker changes. Changes in biomarker levels occurred shortly after exposure to radiation, allowing for better assessment of early signs of damage caused by ionizing radiation before further exposure.

**Fig. 10:**
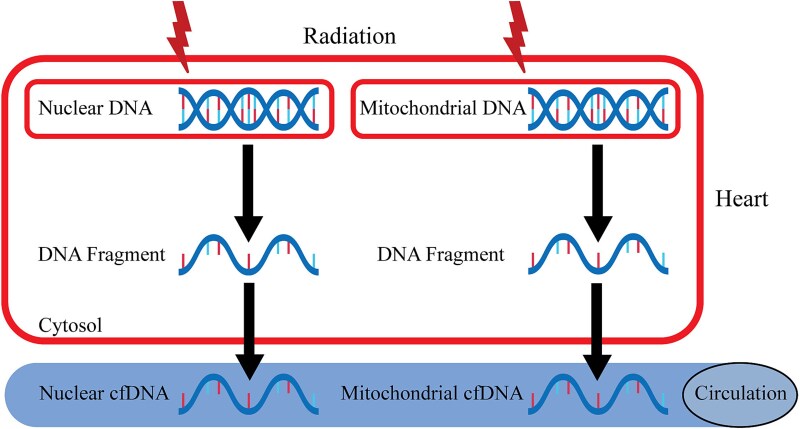
Concept of nuclear DNA and mitochondrial DNA in plasma as potential biomarkers of radiation injury to the heart.

We examined the genomic internals for each mitochondrial-specific rat 12S rRNA and determined regions of those sequences that have no variants in >100 sequenced strains. This experimental approach allowed us to design primers for mitochondrial-specific 12S rRNAs that can be used as analytical tools across multiple rat strains. We evaluated the effect of time and determined that 60 minutes is the earliest time point following radiation of the heart with 3.5 Gy when plasma cell-free nuclear and mitochondrial DNA is increased. The concept underlying the current study is shown in Fig. 10. Future studies are needed to define the time to peak release of cfDNA. Preliminary findings obtained from the new methods established in the present study indicate exposure of male hearts to 50 mGy and female hearts to 3.5 Gy X-radiation increased the levels of nuclear and mitochondrial cfDNA in a time-dependent manner, suggesting a dose–response effect. Male and female rats were studied as both sexes are exposed to medical radiation. The dose of 50 mGy was selected as diagnostic cardiac catheterization in infants of both sexes can expose the heart to this dose of radiation. The dose of 3.5 Gy was selected as being relevant to a single dose delivered during therapeutic radiation where tumors in the thorax such as breast, lung and esophagus are treated with multiple fractions of radiation. As breast cancer is the most common cancer in women in Japan and the USA, we conducted these studies in female rats. Our findings suggest cfDNA may have utility as a biomarker of damage to the heart caused by ionizing radiation in both sexes. Future studies will be needed to determine the dose response in both sexes.

Both necrosis and apoptosis contribute to cfDNA release. When characterizing cfDNA fragments arising from apoptosis and necrosis, apoptotic DNA fragments are significantly shorter (around 166 bp) due to the controlled cleavage during programmed cell death, while necrotic DNA fragments are longer, reflecting the random fragmentation caused by cell membrane disruption. The time interval from the exposure to radiation was a factor affecting cfDNA release and detection. L218/S125, the ratio of the length of the long to short fragment for 12S rRNA, decreased after radiation, suggesting a greater contribution from apoptotic cell death over necrotic cell death. The significant decrease in the ratio observed in Fig. 6, particularly at 6, 30, 72 and again at 96 hours (3.5 Gy) suggests a dramatic increase in DNase activity at these time points. Further studies are needed to evaluate these cfDNA fragment characteristics as a biomarker of radiation injury.

Liquid biopsy, the analysis of cellular components released from dying or damaged cells into the circulation or other bodily fluids, has utility as a biomarker. Our idea is to rapidly quantify an event that happened in intact tissue using plasma, whereas chromosomal aberrations and gammaH2AX measurements are *ex vivo* assays that can take days to complete. Attractive because it is minimally invasive and repeatable, liquid biopsy is gaining traction in early detection and diagnosis, patient stratification, detection of minimal residual disease and prediction of recurrence after primary treatment [37, 38]. The present studies described used blood as the bodily fluid to develop cfDNA as a biomarker of radiation-induced damage. Although the heart is directly exposed to radiation, cfDNA may originate from non-cardiac sources as DNA is expressed in all nucleated cells. During the period of radiation, there is sufficient time for nucleated cells in the blood to transit the coronary vasculature and traverse the systemic and pulmonary circuits. To effectively eliminate DNA from leukocytes in blood, plasma was prepared and two sequential centrifugation steps were included [13]. A portion of the lung may be exposed to radiation. Despite this limitation, the present study shows cfDNAs for *Gapdh,* S125 and L218, can detect early radiation-induced damage and provide insight into radiation toxicity.

Phosphorylation of H2AX is a hallmark of radiation damage to DNA [39]. Following radiation exposure, histone H2AX is rapidly phosphorylated by ATM and/or DNA-PK kinases at or near the vicinity of DNA double-strand break sites to form gammaH2AX [40]. The gammaH2AX assay is widely used as a sensitive molecular marker of DNA damage [41–45]. The timing and magnitude of increased levels of nuclear and mitochondrial cfDNA in the circulation 60 minutes after irradiation with 3.5 Gy in the present study agree with the timing and magnitude of phosphorylation of H2AX after irradiation of human blood lymphocytes with a similar dose of 4.0 Gy [46]. Our findings suggest cfDNA may be developed as a biomarker of radiation-induced damage to complement the gammaH2AX assay.

Cardiac catheterization in infants can expose the heart to doses of radiation around 50 mGy. Median effective radiation dose from cardiac catheterization in patients who developed cancer was 43.0 (0.8–242.3) mSv [47]. Radiation exposure from cardiac catheterization in patients with dilated cardiomyopathy was 68 mSv [48]. Radiation dose to patients from cardiac diagnostic imaging can be as high as 69.9 mSv [49]. Radiation dose to the coronary artery during percutaneous transluminal coronary angioplasty was 60.6 mGy [50]. The present study shows exposure of the rat heart to 50 mGy increased cfDNA levels in plasma. Further studies are needed to determine whether a dose of 50 mGy increases cfDNA levels in patients undergoing cardiac procedures.

cfDNA present in blood can originate from passive release from apoptotic [51] and necrotic cells [52] and active release from living cells [53]. Physiological variables such as body mass index, menstruation, hypertension, circadian rhythm, stress, biological sex and age can influence cfDNA levels in blood [54]. Cancer, exercise and smoking can affect cfDNA levels in blood [54]. Systemic inflammation induces release of cell-free DNA from hematopoietic and parenchymal cells in mice and humans [55]. Patients who require medical radiation may have pre-existing co-morbidities such as hypertension with and without inflammation that influence cfDNA levels. Thus, release of cfDNA from cells into the circulation is not specific to radiation. The experimental approach in the present study addressed this issue. Medical radiation is an elective procedure. In this setting, blood is obtained before and after exposure to radiation and levels of cfDNA measured. This aspect of the experimental design will account for factors that can affect cfDNA levels before radiation exposure such as inflammation from the impact of radiation exposure by itself because each time a measurement of cfDNA is required, the patient serves as their own control to account for blood levels prior to radiation. Thus, cfDNA has potential for advancement as a blood-based biomarker of radiation damage to the heart in clinical settings.

Previous studies have measured ALU retrotransposons as a marker of circulating cfDNA. Alu repeat sequences account for at least 10% of the whole genome and are normally confined within cells. The earliest response to radiation was observed at 24 hours after exposure of the total body to 1 Gy using ALU sequences [56]. Doses relevant to diagnostic radiation were not measured in this study using ALU sequences or in previous studies [57, 58]. Exposure of the whole body to 3 Gy from X-radiation increases in cfDNA in the urine of rats at 6 hours above values in sham-irradiated rats [27]. Future studies are needed to evaluate whether urine samples collected from rats before and after exposure of the heart to medical radiation contain nuclear and mitochondrial cfDNA using the method we have established.

## CONCLUSION

We have demonstrated our technical capability to measure nuclear and mitochondrial cfDNA in rat plasma and show increases resulting from exposure of the heart to doses of radiation used for diagnostic and therapeutic procedures. cfDNA has the potential to advance as a biomarker of exposure to diagnostic and therapeutic doses of radiation in patients. Further research is needed to explore the effects of radiation on cfDNA in other organs that may be incidentally exposed. The method we are developing uses a simple RT-PCR assay where results are obtained within hours. Validation of cfDNA as a biomarker of radiation damage will help improve our understanding of the biology of medical radiation. cfDNA may be combined with mass spectrometry, second-generation sequencing and single-nucleotide polymorphisms [7] to identify the underlying factors that result in radiogenic disease.

The data underlying this article will be shared on reasonable request to the corresponding author.
